# Housing, income support and mental health: Points of disconnection

**DOI:** 10.1186/1478-4505-5-14

**Published:** 2007-12-12

**Authors:** Cheryl Forchuk, Libbey Joplin, Ruth Schofield, Rick Csiernik, Carolyne Gorlick, Katherine Turner

**Affiliations:** 1 School of Nursing, University of Western Ontario, London, Ontario, Canada; 2 Mental Health Nursing Research Alliance, Lawson Health Research Institute, London, Ontario, Canada; 3 Faculty of Health Sciences, McMaster University, Hamilton, Ontario, Canada; 4 School of Social Work, University of Western Ontario, London, Ontario, Canada; 5 Community Futures, Industry Canada-FedNor, Canada

## Abstract

There exists a disconnection between evolving policies in the policy arenas of mental health, housing, and income support in Canada. One of the complexities associated with analysing the intersection of these policies is that federal, provincial, and municipal level policies are involved. Canada is one of the few developed countries without a national mental health policy and because of the federal policy reforms of the 1970s, the provincial governments now oversee the process of deinstitutionalization from the hospital to the community level. During this same period the availability of affordable housing has decreased as responsibility for social housing has been transfered from the federal government to the provincial and/or municipal levels of government. Canada also stands alone in terms of being a developed nation without national housing policy instead what is considered "affordable" housing is partially dependant upon individuals' personal economic resources. As well, over the past decade rates of income supports have also been reduced. Psychiatric survivors have long been identified as being at risk for homelessness, with the disconnection existing between housing, income and mental health policies and the lack of a national policy in any of these policies areas further contributing to this risk.

## Background

There exists a well known relationship between the presence of a mental illness and increased rates of poverty and homelessness. However, the exact dynamics of this relationship has remained somewhat unclear especially in the context of present day Ontario. While early literature suggested that these increased rates of homelessness and unstable housing were due to the presence of a mental illness [1,2]. More recent literature has suggested that these increased rates are more likely the result of an overall lack of affordable housing [3-5]. In Canada, the lack of national housing, mental health and income policies have further added to the basic problems related to the issues of housing and mental health. At the provincial level in Ontario, an examination of the policies pertaining to the issues of housing development, income support and mental health brings to light a large area of disconnect existing between these policy realms. The purpose of this paper is to answer, by means of an analysis of the available research literature, the questions; what are these areas of policy disconnect and what is their impact on those who rely on these policies?

### Housing Development

Under section 92 of the *Constitution Act*, 1867, responsibility for the development of social housing rests with the provincial government however the federal government has the ability to unilaterally initiate social housing programs under the Peace, Order, and Good Government Clause of the *Act*, as long as these programs are determined by the courts to be in the national interest. Until the 1990s the federal government was involved with a number of different partners, including the provincial governments, in the development of social housing stock. However, at that time, the federal government transferred administrative responsibility for the majority of this housing stock to the provincial governments and has since proceeded to further cut the funding of programs supporting the development of new housing stock. Presently, the federal government continues to have an administrative role only in the federal co-operative social housing sector.

After the 1995 election of Mike Harris' ideologically and politically conservative government, the province of Ontario transferred ownership of administrative responsibility for the existing social housing stock to newly created municipal service managers. A one-time allocation of funds and a substantial body of operational regulations accompanied this transfer. At the same time, the Harris provincial government cancelled the previously existing housing development agreements and signalled that any subsequent affordable housing developments would be the responsibility of the private sector.

The definition of affordable housing, according to the Canada Mortgage and Housing Corporation (CMHC) varies with the number of bedrooms, number of levels, and the location of a given home within Ontario. For instance, according to the CMHC, for a one bedroom, one level apartment in the city of London Ontario to be considered affordable, the rent must be below the maximum of $700 a month while an apartment having the same number of levels and bedrooms in the city of North Perth must be below a maximum of $575 a month and in the city of Toronto, must be below the a maximum of $990 a month to be considered affordable. Between 1995 and 2002 almost no new affordable housing developments arose in the province of Ontario due in part to the general reluctance of the private housing developers to invest in the development of new affordable housing, a venture they considered to be somewhat less than attractive [6].

Throughout this period, community level pressure for the creation of new affordable housing developments escalated, fuelled by increasing levels of homelessness in cities across the country [7]. This has led to renewed federal interest in the development of affordable housing stock. As a result, in 2001 the federal government initiated a federal-provincial partnership regarding the development of affordable housing and proceeded to negotiate agreements on a province-by-province basis. The agreements were intended to leverage matching provincial dollars of $25,000 per unit to provide the upfront capital funds necessary for housing development.

In Ontario the provincial government signed an agreement with the federal government in 2002, in which the Ontario government would provide a provincial sales tax rebate of $2,000 per unit while the participating municipalities and the participating housing developers would provide an additional $2,000 per unit. In 2004, a number of housing developments were proposed but had yet to be implemented despite the election of an Ontario liberal government in October 2003 that had expressed a commitment to the development of new subsidized and supportive housing units within the province, nor has it been an issue discussed by the governing Liberals as part of the forthcoming fall 2007 provincial election.

### Income Supports

Individuals with mental illnesses in Ontario are most likely to obtain public income support through either the Ontario Works or the Ontario Disability Support Program. Other sources of income support for such individuals first require a period of employment, requirements that persons with serious mental illnesses may have difficulty achieving. For example, to obtain income support through the Canada Pension Plan (Disability) (CPP (D)) a person must be employed for at least four of the previous six years, a criterion that individuals with serious mental illnesses often have difficulty meeting. As well, the CPP (D) requires a potential recipient to have an "ongoing" illness which serves as a barrier for those with mental health issues given that many mental illnesses are episodic and not ongoing in nature. Similarly, those individuals with mental illnesses would be eligible for private insurance coverage only if they became ill while employed or if the Workplace Safety Inspection Board (WSIB) determined that the employee suffered an injury while at work. However, it would be very unlikely for a person suffering a mental illness to be eligible for such coverage given that the development of mental illnesses have rarely been linked to workplace injuries.

#### i) Ontario Works (OW)

In May 1998, the *Ontario Works Act *replaced the existing General Welfare Assistance program though no increase in actual financial support accompanied this change in program direction. Currently the amount of income support meant to meet the basic needs of a single person with no dependents is $195.00 per month, a number which increases to the sum of $512.00 per month if the person has a partner and dependent child over the age of 13.

The Harris provincial government was very clear in that Ontario Works was to serve as a labour market adjustment program with the provision of employment assistance as its primary goal. This program therefore was intended only to provide temporary financial assistance to people in need, it was not conceptualized as a social assistance program. The government believed that previous welfare rates had been so generous that they failed to encourage self-reliance and instead gave rise to widespread dependency on welfare thus serving to discourage people from seeking employment in Canada's most prosperous province. In accordance with the government's interpretation of social assistance programs, a mandatory welfare-to-work program was instituted in the province along with a diligent crack down upon any perceived systematic or individual fraud. The fundamental philosophy underlying the Ontario Works program was that the shortest route to paid employment must be the overriding principle of all of the program's activities [8]. The program's eligibility rules were designed to encourage individuals in financial need to seek employment as a first resort and to seek social assistance only when all other resources and possibilities had been exhausted. It was believed that these rules would help to ensure that social assistance funds were given to those considered to be the most in need of them: the deserving poor.

A range of employment assistance activities were introduced including programs to provide those participants who were not job-ready with employable skills, programs to assist participants who were job ready in the process of finding paid employment, and programs designed to support participants in finding the shortest route possible to paid employment through means of community participation and various employment measures. All persons involved with Ontario Works are required to participate in one or more employment assistance programs to be considered eligible for financial assistance, unless these requirements are deferred under special circumstances [8]. This stipulation, like many other Ontario Works stipulations, was intended to move people towards becoming more self-reliant. The *Ontario Works Act *provides a legislative framework that mandates local delivery agents to offer specific types of assistance including referrals to basic education opportunities where appropriate, making available skills training initiatives to assist participants in becoming job-ready and to help put each participant on the shortest route possible to the goal of paid employment. This training is intended to be job-specific, with the goal of providing an entry into or a return to employment in the shortest amount of time possible.

The *Act *does provide for basic financial assistance to be paid to persons who meet the eligibility requirements [9]. Basic financial assistance under this Act includes:

• income assistance provided for the purposes of basic needs and shelter;

• benefits as prescribed in the regulations, e.g. prescription drugs;

• emergency assistance provided to help with basic needs and shelter on an emergency basis.

The *Ontario Works Act *also provides employment assistance to recipients based on their individual skills, experience and circumstances [9]. Employment assistance offered under this Act includes:

• *community participation *in activities that allow people to contribute to their community and to improve their level of employability; and *other employment measures *including:

• job search support services, including employment resource centres

• literacy screening tests

• literacy assessment, literacy training or both

• other basic education and job-specific skills training sessions, including sessions pertaining to life skills

• employment placements

• education or training programs

• self-employment activities

• supports pertaining to self-employment

• substance abuse recovery programs and

• LEAP – Learning, Earning, and Parenting, a program designed to help teen parents receiving social assistance to finish high school, to learn more about what it takes to be good parents and most importantly how to get a job as quickly as possible.

In May 2001, the government announced a five-point action plan designed to make Ontario Works an even more responsive program including:

• following through on the government's commitment to the practice of double placements in order to provide more people with the opportunity to gain the job-related experience they need to both find and keep a job

• providing additional supports for people on welfare who face significant employment barriers

• mandatory literacy testing and training to help people who can't read and write well enough to both find and keep a job to break through the literacy barrier and overcome these obstacles

• offer advanced caseworker training in order to provide them with the skills that they need to better assist those facing employment barriers take the steps required to move from welfare to work

• mandatory addiction treatment to help people overcome addictions serving as an obstacle to employment

The goals of Ontario Works, as outlined in the *Ontario Works Act*, is the establishment of a program that recognizes individual responsibility and promotes self-reliance through employment, that provides temporary financial assistance to those most in need while they satisfy obligations to become and stay employed, and that holds the government sufficiently accountable to the taxpayers of Ontario [10].

#### ii) Ontario Disability Support Program (ODSP)

The Ontario Disability Support Program is an adjunct to Ontario Works as indicated by the transitional provisions listed under Schedule D of the *Social Reform Act *of 1997. The intent of this program is to provide both income and employment supports to persons with disabilities and their dependants who meet the strict criteria stipulated by the *Act*. According to the *ODSP Act*, a person is considered disabled if:

a) the person has a substantial physical or mental impairment that is continuous or recurrent and expected to last one year or more;

b) the direct and cumulative effect of the impairment on the person's ability to attend to his or her personal care, function in the community and function in a workplace, results in a substantial restriction in one or more of these activities of daily living; and,

c) the impairment and its likely duration and the resulting restrictions it places on the person's activities of daily living have been verified by a person having the prescribed qualifications [11].

While the ODSP Act does not precisely define what is meant by the term 'substantial', given the context in which the term is used, it can be inferred to mean a disability that gives rise to a number of obstacles in one's life that can only be overcome with government assistance.

The application process entails completion of a health status report, activities of daily living report, a medical consent form and a self report. However, single status persons are not eligible for the program if they have over $5000 in liquid assets, cash, Registered Retirement Savings, or insurance policies while those persons with a partner may have up to $7,500 in assets and still qualify for the program [12]. In addition, in direct opposition to Human Rights legislation, persons are deemed ineligible for ODSP if they are found to be dependent upon or addicted to alcohol or any other psychoactive drug unless otherwise authorized [11].

Becoming qualified for ODSP has become extraordinarily difficult since its evolution from the former GAINS-D (Guaranteed Annual Income Supplement for the Disabled) program. In 1998, there were 189,442 active cases on file, three quarters of which pertained to single status individuals. By 2003, the caseload had grown by 11,718 to a total of 201,160, a rate of just slightly over one per cent per year [13].

While the income support provided by ODSP is substantially greater than that provided by Ontario Works, it still provides recipients with an income significantly below any of the established poverty line levels. In 2002, The National Council of Welfare [13] conducted an analysis comparing income support benefits in constant dollars and found that a single individual receiving ODSP that year could have obtained a maximum amount of $11,466, nearly double that of an individual on Ontario Works at the time. However, the equivalent income in 1997 when the legislation was first introduced was $12,682 while the equivalent income in 1992 would have been $13,449. Thus a single person without any substantial assets and who, under government policy, is considered to be permanently unemployable has seen the amount of income support they're eligible to receive decreased by 10.6% since the new legislation was passed and has seen the amount of real dollars support deceased by 17.7% in the course of a single decade. In this same time period, income support for a single employable person receiving Ontario Works benefits fell in the province by nearly half from $9,741 per year to $6,623, a pattern that has seen itself repeated in every Canadian province and territory.

### Mental Health Care: Deinstitutionalization

Bachrach [15] defined deinstitutionalization as a process having two main elements: the avoidance of traditional institutions/hospitals, and the concurrent expansion of community based facilities (p. 573). The "deinstitutionalization" of individuals diagnosed with a mental illness has been a process that has been evolving for decades. Goldman and Mossissey [16] described cycles of institutional reforms such as the early 19^th ^century introduction of moral treatment through the use of asylums; the mental health hygiene movement and psychopathic hospitals; and the mid 20^th ^century community focused mental health movement. They argue that the fourth cycle in this evolutionary process is the establishment of a broad network of both mental health and social welfare services. However, according to Goldman and Mossissey the

"failure to address the basic social problems themselves has resulted in a repeating cycle of policies which only partly accomplish the goals of their activist proponents" (p. 727).

Sussman [17] described the evolution of psychiatric facilities across Canada as a process that has occurred with little in the way of communication or collaboration between the provinces. Sussman also differentiated between the process of "deinstitutionalisation" and the process of "dehospitalization" and discovered that, many so-called "community" programs were in actuality simply smaller institutions.

The movement associated with making the community the focus of care instead of psychiatric hospitals began with the introduction of effective anti-psychotic drugs after the 1950's [18]. In Ontario, the total number of patients in provincial psychiatric hospitals peaked in 1959–1961, with the largest decrease occurring between 1965–1968; In 1960, there were 19,501 hospitalized patients in Ontario while in 1982, there were only 4,514 [19].

In recent decades there have been a number of provincial discussion papers and policy documents written that advocate for mental health policy reform. The Ontario branch of the Canadian Mental Health Association [20] has noted that 20 such documents and papers have been produced in as many years. The process of deinstitutionalization and the corresponding movement of the focus of care to the community has served as the predominant focus of these documents and papers. Since the "Putting People First" report of 1993 [21], this proposed recommendation has included an explicit shift in funding such that 60% of the funding for mental health services would be distributed to community-based programs while the remaining 40% would be distributed to hospital-based programs. This recommendation therefore was in essence, the mirror image of the funding situations existing at that time.

In 1999, a provincial health care restructuring commission recommended that the provincial psychiatric hospitals (PPHs) be transferred by the provinces to local community service centres and/or to community hospitals [22]. The document called for a reduction in hospital beds and stated that "the proposed hospital bed targets are achievable once the appropriate community services and supports are in place to reduce reliance on institutional care, especially PPHs, and dramatically reduce the need for hospital-based treatment services" [23]. The report further states that, "in 1995/96 there were 2,900 mental health beds in PPHs. The number of beds that will be available post -restructuring [2003] is estimated at 1,767 beds, resulting in a decrease of 1,133 beds (39 per cent decrease)." (p. 6). All of these decreases in the number of hospitalized patients occurred as the province's overall population steadily grew with each passing year.

Despite this dramatic decrease in the funding of psychiatric hospitals and the reduction of the number of available beds, a concurrent shift of resources to community-based mental health programs has not occurred. In addition, given that psychiatric hospitals provided patients with housing as well as mental health services, the need for new homes has increased as the process of dehospitalization and bed reductions have also increased.

There has been no corresponding increase in the funding of community mental health programs since 1993, despite the larger numbers of people seeking these community based services. With the recent change in government to the Liberal party lead by Dalton McGinty there was a promise of increased community mental health funding and after years of no increases, some increases have been made since 2004 in key areas such as crisis response, early intervention for psychosis and case management.

*The rhetoric of mental health reform policy *paper [24] and the Putting People First policy document both recognized the need to better take into account recognized health determinants and to provide a more comprehensive vision of health rather than narrowly focusing upon the alleviation of illness in the delivery of mental health services. In 1998, the Ministry of Health policy document entitled *2000 and Beyond : Strengthening Ontario's Mental Health System *[25] recognized that one of the principles guiding the reform of mental health policies should be the adoption of a holistic approach that addresses broader determinants of health such as housing and income support. Unfortunately in the more recent policy document *Making It Happen – Implementation Plan for Mental Health Reform *this holistic principle was rejected as a guiding principle underlying mental health policy reform while at the same time, the document stated that increasing the quality of life of the mental ill must be a goal of all subsequent reforms. The question that then arises is how can the government expect the quality of life of the mentally ill to improve if such well established determinants of health, as housing and poverty are not taken into account in the relevant policies?

### Implications

There appears to be a large area of disconnect existing between the evolving policies regarding housing, income support and mental health care. The move to shift the focus of care of psychiatric survivors to community-based centres away from the hospital and institutional setting occurred while the availability of affordable housing was experiencing a decrease. The increased restrictions placed on income support further reduced the availability of housing that could be considered affordable to this population. The disconnection existing between these policy areas has created a situation which has increased an already vulnerable population's risk of being reducing to a state of homelessness. Since these policy changes have occurred simultaneously throughout multiple policy sectors, the resulting areas of disconnect are often poorly understood even by service providers within each policy sector. Solutions to the problems resulting from the existing areas of disconnect can only arise by re-establishing and strengthening the connections existing between these diverse policy arenas.

What is often neglected in the discussion of mental health policy and practice is the actual voice of the consumer or recipients of these services. Consumers of these services have described the disconnect existing at the mental health service provider level and client level as "resembling a false ploy doubling as an art form in practice." In some circumstances, no matter how unhealthy a situation becomes, the service provider's credo remains essentially "don't worry dear, we have everything under control," even when circumstances clearly have past beyond their control. Hence, the creation of policy disconnection is also achieved at the interpersonal level when fending off a complainant's concerns. Given the disconnection existing between evolving policies in the areas of mental health, income support and housing, some psychiatric consumers may be much more accurately described as being system survivors.

## Conclusion

Health care and social service professionals as well as policy makers need to understand and acknowledge the areas of disconnect existing between the various policies related to mental health, income maintenance and housing. Clearly, there is a role for advocacy to increase community awareness and to promote system level change. However, proper acknowledgement of the disconnection includes working on a personal level with clients and avoiding the use of therapeutic euphemisms or pathologizing very real poverty related fears. On the whole, the disconnection existing between housing policy, income support policies, and mental health policies adversely impacts the lives of many of those these policies were meant to improve. It is this "disconnect" that causes many of these individuals to feel as though they've been disregarded by society, to be harmed even further, and to minimize the experiences of those with mental illnesses. These disconnections bind people while holding them back and erodes their sense of dignity, worth, stability and security. It effectively serves to re-victimize the already aggrieved, disenfranchised and at-risk and it is our hope that through means of reforming the aforementioned practices, programs, and policies, we can reverse this trend and better serve those individuals these programs we intended to benefit.
